# Depression and anxiety are increased in autism and ADHD: Evidence from a young adult community‐based sample

**DOI:** 10.1002/jcv2.70003

**Published:** 2025-03-26

**Authors:** Simone Capp, Aoibhe De Burca, Ümit Aydin, Jessica Agnew‐Blais, Alexandra Lautarescu, Angelica Ronald, Francesca Happé, Gráinne McLoughlin

**Affiliations:** ^1^ Social, Genetic & Developmental Psychiatry Centre Institute of Psychiatry, Psychology & Neuroscience King's College London London UK; ^2^ School of Psychology & Clinical Language Sciences University of Reading Reading UK; ^3^ School of Biological & Behavioural Sciences Queen Mary University of London London UK; ^4^ Institute of Psychiatry, Psychology & Neuroscience King's College London London UK; ^5^ School of Psychology University of Surrey Guildford UK

**Keywords:** ADHD, anxiety, autism, depression, mental health, neurodivergent traits, young adult

## Abstract

**Background:**

Autism and attention‐deficit/hyperactivity disorder (ADHD) overlap to a considerable degree and have been associated with mental health difficulties, yet there is limited research on this relationship. Young adulthood is a time of heightened risk for mental health problems in general. The risk may be greater for individuals with these conditions, for whom societal demands tied to this transitional time may heighten the impact of internalising behaviours. Elucidating the relationships between neurodevelopmental differences and vulnerability to psychopathology may inform future adaptations for specialised support.

**Methods:**

This study explored whether autistic and ADHD traits and their interaction were associated with symptoms of depression and anxiety as well as meeting diagnostic criteria for internalising disorders in a sample of 556 young‐adult twins (mean age 22 years 5 months, 52% female), controlling for sex, age, cognitive ability, and parental socioeconomic status. Four participant groups were created based on traits assessed in young adulthood: high autistic traits, high ADHD traits, high autistic *and* ADHD traits, and low ADHD *and* autistic traits.

**Results:**

High autistic and ADHD traits were independently associated with higher self‐reported depression and anxiety symptoms and likelihood of meeting diagnostic thresholds for an anxiety or low mood disorder. While co‐occurrence of autism and ADHD exhibited the greatest risk for mental health challenges, no evidence was found for interaction effects between these traits at stringent corrected thresholds. Females with high levels of autistic traits exhibited particularly high risk for concomitant psychopathology.

**Conclusions:**

Our findings suggest that those with high levels of autistic and/or ADHD traits may require individualised care strategies, in light of the complex interplay between traits of neurodivergence and mental health outcomes. Future research may explore the efficacy of psychoeducation and specific adaptations to established therapeutic interventions needed to optimise outcomes for adults with these conditions.

## Graphical abstract

In a large community sample of young adults, we aimed to investigate the relationship between autism, ADHD and how they interact and symptoms of anxiety and depression. We found that both conditions increased the likelihood of reporting these symptoms but that the effects of autism and ADHD on mental health challenges were independent with limited evidence of an interaction between them. This means that the impact of autism remains the same whether or not ADHD is present and vice versa. However, it is the case that having both conditions increases the likelihood of mental health challenges.

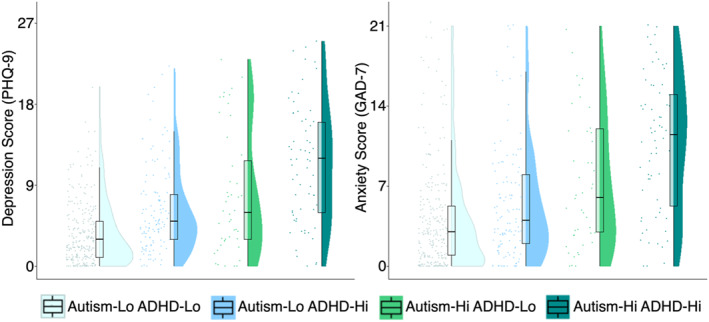

**Key Points**

- Traits characteristic of autism and ADHD, as well as official diagnoses, overlap considerably and are associated with increased risk of developing mental health problems
- Autism and ADHD are understudied in adults, particularly their co‐occurrence, despite the societal demands of early adulthood placing additional strain on mental health generally which may represent a heightened risk for individuals with these conditions
- Young adults with high autistic or ADHD traits, and especially both, reported greater depression and anxiety symptoms and were more likely to meet diagnostic criteria for internalising disorders, with females with high autistic traits reporting higher symptom levels in particular
- Adults with high levels of autistic and/or ADHD traits may benefit from increased availability of specialised support and psychoeducational material targeting mental health difficulties

## INTRODUCTION

Early adulthood can be a period of significant adjustment, with potentially heightened challenges for individuals with neurodevelopmental differences (Crane et al., 2019). Multiple lines of evidence indicate that adults with autism spectrum conditions (henceforth ‘autism’) or attention‐deficit/hyperactivity disorder (ADHD) experience elevated rates of mental health difficulties compared to neurotypical adults (Agnew‐Blais et al., 2018; Chen et al., 2018; Fayyad et al., 2007; Hollocks et al., 2019; Lai et al., 2019; Lugo‐Marín et al., 2019). Moreover, this association holds true not only for diagnosed, but also subclinical cases (Garcha & Smith, 2024).

While autism and ADHD are distinct conditions, they frequently co‐occur, a phenomenon supported by genetic evidence suggesting a broadly overlapping aetiology (Larsson et al., 2012; Lundström et al., 2012; Robinson et al., 2011; Ronald et al., 2006; Stergiakouli et al., 2015). This intersection of autistic and ADHD traits persists throughout the lifespan (Hartman et al., 2016; Jensen & Steinhausen, 2015; Rong et al., 2021), partly due to shared genetic influences (Riglin, Leppert, et al., 2021; Ronald et al., 2014). However, despite the lifelong impact of these conditions, research has predominantly focused on childhood and adolescence, leaving a gap in our understanding of how autism and ADHD co‐occur and affect individuals in adulthood (Hartman et al., 2016; Mason et al., 2022).

Given that autistic traits have been widely associated with a heightened risk for mental health conditions in adulthood (Hollocks et al., 2019; Kanne et al., 2009; Lai et al., 2019; Lugo‐Marín et al., 2019; Stice & Lavner, 2019), as is the case for traits of ADHD (Chen et al., 2018; Fayyad et al., 2007; Naya et al., 2021), this naturally raises the question of whether their overlap increases vulnerability to psychopathology. Existing studies in this area have shown robust associations between diagnosed autism/ADHD and internalising disorders, revealing specific patterns of psychiatric comorbidity and sex differences, while also highlighting shared vulnerabilities (Joshi et al., 2020; Pehlivanidis et al., 2020; Solberg et al., 2019). Beyond a high prevalence of co‐occurring anxiety and depression in general, findings indicate that individuals with both autism and ADHD diagnoses face the highest risk across a range of psychiatric disorders (Chen et al., 2015).

The strength of this association therefore underscores the need for a ‘lifespan approach’ that extends research beyond childhood and adolescence (Hartman et al., 2016). Lundström et al. (2011) found that autistic traits in adulthood, when examined alongside ADHD traits, showed a consistent increase in the risk for general psychopathology. In a similar vein, Uljarević et al. (2016) posit that internalising problems compound core and co‐occurring symptoms in autistic adults, driving an upward trend of anxiety and depression wherein autistic traits are predictive of symptom severity. Davis et al. (2011) echo this cyclical perspective, observing similarly increased anxiety in adulthood. While bidirectional relationships between psychopathology and adult ADHD are theorised (Katzman et al., 2017), research has primarily focused on aetiology. Shifting attention to the long‐term course and impact of neurodivergent traits could enhance our understanding of their relationship with mental health in young adulthood (Hartman et al., 2016). The presence of multiple co‐occurring conditions in childhood appears to predict the persistence of ADHD symptoms in adulthood (Riglin et al., 2016) and the age of onset for both ADHD and autism symptoms appears more variable than previously believed (Riglin, Wootton, et al., 2021).

Hartman et al. (2016) hypothesise that behaviours relevant to autism and ADHD co‐occur most in emerging adulthood, with its increasing demands on executive function skills, social adaptation challenges, and expectations of independence. They further argue that comparatively lower co‐occurrence rates in childhood may be explained by more diffuse symptom presentations. The new socio‐occupational, personal, and financial roles that characterise the transition from childhood and adolescence to young adulthood (Riglin et al., 2023) likely present unique challenges to neurodivergent individuals, the impact of which has yet to be elucidated. Despite growing interest in this area, the element of reciprocity remains underexplored; for instance, in a highly informative longitudinal study exploring the mental health trajectory of children with autism or ADHD, Orm et al. (2021) acknowledge the considerable overlap between these conditions and its potentially influential role, yet nonetheless neglected to include a dual‐diagnosis group, or any other measure of interaction. As such, there remains an ‘appreciable gap’ in the literature on neurodevelopmental disorders and psychiatric comorbidities into emerging adulthood (Turner, 2019).

Contributing to this gap, Hollocks et al. (2019) note an overreliance on clinical samples in their systematic review of psychopathology in autistic adults. In their call for a greater focus on adulthood, they additionally recommend the inclusion of community‐based samples to reduce heterogeneity and bias, particularly with respect to complex psychiatric presentations. This is reiterated by several researchers who emphasise that those presenting in clinical contexts likely represent more extreme or archetypal cases, whereas, in reality, mixed trait profiles are more common (Grzadzinski et al., 2016; Hartman et al., 2016; Miodovnik et al., 2015). A traits‐based, community‐sample approach therefore offers some distinct advantages, including representation of subclinical cases where individuals may nonetheless demonstrate subtle differences in functioning and increased vulnerability to mental health difficulties (Horwitz et al., 2020).

In the present study, we aimed to examine how high traits of autism, ADHD, or both associate with anxiety and depression in a community‐based sample of young adults. For this purpose, we analysed existing data from a large UK cohort of twins collected during young adulthood, the ‘in‐between’ phase bridging adolescence and fully‐fledged adulthood, a time of heightened risk of mental health difficulties (Kessler et al., 2005; Patel et al., 2007). This dataset represents a non‐clinical sample that is community based but enriched for traits of ADHD and autism via several differing recruitment pathways. As such, we were able to identify individuals displaying high levels of autistic traits, ADHD traits, both, or neither. The sample offers several advantages, notably the availability of both self‐report and interview measures which may minimise bias of discrepancies between assessment types, especially due to masking (Hollocks et al., 2019; Lai et al., 2017; Pearl et al., 2017).

We therefore sought to investigate how traits of autism and ADHD associated with self‐reported symptoms of depression and anxiety, hypothesising that high levels of autistic traits, ADHD traits, and a combination of both would predict higher levels of symptoms. Similarly, we hypothesised that traits of autism and ADHD would be associated with an increased likelihood of meeting diagnostic criteria for low mood or anxiety disorders. Given the paucity of prior research in this area, we made no specific predictions as to whether the interaction between autistic and ADHD traits plays a significant role in young adult mental health.

## METHODS

Analyses reported here form part of a larger project for which aims and analyses have been pre‐registered (https://osf.io/cmxu8).

### Participants

Data from this study come from the Individual Differences in EEG in young Adults Study (IDEAS, described in Capp et al., 2023). Between February 2017 to May 2019, 556 young adults participated in IDEAS, when they were aged 20–25 years (*M* = 22 years 5 months, *SD* = 12 months; 52% female).

Participants in this study were all part of the Twins Early Development Study (TEDS) cohort, a community sample of twins born in England and Wales between 1994 and 1996 (Haworth et al., 2013). Although IDEAS is a community‐based sample, it is enriched for ADHD and autism: participants were selected based on child and adolescent ADHD and autistic trait scores. All participants had an estimated IQ of 70 or above. Further information is provided in Supporting Information S1: S1.

### Procedure

The King's College London Psychiatry, Nursing and Midwifery Research Ethics Subcommittee granted ethical approval for the study (RESCMR‐16/17‐2673).

Participants completed online questionnaires prior to completing an in‐person assessment session arranged with the research team. In‐person sessions were carried out in the research centre or in participants' homes and lasted approximately 2.5–4.5 h, during which participants provided written informed consent, completed interviews and behavioural assessments, and undertook computerised cognitive tasks while wearing a portable EEG device. Participants were compensated for travel expenses and received a voucher.

### Measures

All self‐report measures included here, as well as tests of cognitive ability, were completed as part of the online questionnaires hosted on Qualtrics (Hollocks et al., 2019; Kanne et al., 2009; Stice & Lavner, 2019) and took around an hour to complete. Observer‐rated and interview measures were completed during in‐person testing sessions facilitated by a trained researcher. Core measures are summarised below, and Supporting Information S1: S2 contains a detailed description of the measures as well as additional information about measures of cognitive ability and parental socioeconomic status (SES).

### Grouping measures

#### Autism Diagnostic Observation Schedule (ADOS‐2)

The ADOS‐2 is a gold‐standard semi‐structured observational assessment used for the diagnosis of autism spectrum conditions (Lord et al., 2012). Participants completed Module 4 of the ADOS‐2, which includes a range of activities and interview questions. Calibrated Severity Scores (CSS) were generated as standard (Hus & Lord, 2014), with a possible range of 1–10, where scores of 4 or higher indicate behaviours and characteristics consistent with an autism spectrum diagnosis.

#### Social Responsiveness Scale‐2 (SRS‐2)

The SRS‐2 adult self‐report questionnaire is a 65‐item measure that assesses social and behavioural autism characteristics (Constantino & Gruber, 2012). Participants rate the applicability of statements (e.g. “I am much more uncomfortable in social situations than when I am by myself”) using a 4‐point Likert scale and raw sum scores range from 0 to 195. Sum scores of 68 (corresponding to standardised T‐scores of 60) or higher are considered to identify participants with significant autism‐related social and behavioural difficulties which have a mild‐severe impact on everyday social interactions (Constantino & Gruber, 2012).

#### Diagnostic Interview for ADHD in Adults 2.0 (DIVA 2.0)

The DIVA 2.0 is a semi‐structured diagnostic interview assessing adult ADHD (Kooij & Francken, 2010). During the interview, participants are asked about their experience of ADHD symptoms, both currently and during childhood (e.g. “Do you often find it difficult to sustain your attention on tasks?”). The DIVA 2.0 interview was used to identify participants likely to meet DSM‐5 diagnostic criteria for adult ADHD. To meet diagnostic criteria, participants are required to endorse five or more symptoms of inattention or hyperactivity/impulsivity, report that at least some symptoms began in childhood, and feel that these difficulties have negatively impacted more than one area of their life (American Psychiatric Association, 2013).

#### Barkley Adult ADHD Rating Scale (BAARS‐IV)

The BAARS‐IV (Barkley, 2011) self‐report questionnaire includes 27 items (e.g. “Difficulty sustaining my attention in tasks or fun activities”) assessing characteristics associated with ADHD using a 4‐point Likert scale. Total sum scores range from 18 to 72, where higher scores indicate greater ADHD‐related traits and behaviours. A cut‐off score of 39 or above was used to identify those with mild‐marked ADHD symptoms. This cut‐off was found to represent scores in the 93^rd^ percentile in a general population sample of adults (Barkley, 2011).

#### Grouping variables

Scores from the ADOS‐2, SRS‐2, DIVA 2.0, and BAARS‐IV were used to create two overlapping grouping variables to identify those with high levels of autistic (Autism‐Hi) and/or ADHD traits (ADHD‐Hi).

Participants were deemed to show high levels of autistic traits (Autism‐Hi) if they demonstrated an ADOS‐2 Calibrated Severity Score (CSS) of four or above and/or an SRS‐2 raw score of 68 or more (with a T‐score cut‐off of 60). This group represents individuals likely to meet the criteria for an autism spectrum condition diagnosis (Autism‐Hi). Participants not meeting either of these criteria were considered to exhibit low levels of autistic traits (Autism‐Lo).

Similarly, participants were regarded as showing high levels of ADHD traits if they met DSM‐5 adult ADHD criteria using the DIVA 2.0 and/or if their total BAARS‐IV score was 39 or higher, representing individuals likely to meet diagnostic thresholds for adult ADHD (ADHD‐Hi). Participants not meeting either of these criteria were considered to have low levels of ADHD traits (ADHD‐Lo).

These two grouping variables were not mutually exclusive, thus resulting in four combined‐trait groups in total: Autism‐Lo ADHD‐Lo (*n* = 296, 66.5% female), Autism‐Lo ADHD‐Hi (*n* = 131, 44.3% female), Autism‐Hi ADHD‐Lo (*n* = 55, 34.6% female), and Autism‐Hi ADHD‐Hi (*n* = 72, 45.8% female).

### Mental health measures

#### Patient Health Questionnaire (PHQ‐9)

The PHQ‐9 is a commonly used self‐report screening questionnaire for depression symptoms (Kroenke et al., 2001; Kroenke & Spitzer, 2002) comprising nine items for which participants are asked to rate how often a statement (e.g. “Little interest or pleasure in doing things”) has applied for them in the previous 2 weeks on a 4‐point Likert scale, with total scores ranging from 0 to 27 wherein higher scores indicate higher levels of depressive symptoms.

#### Generalised Anxiety Disorder Assessment (GAD‐7)

The GAD‐7 is a self‐report screening questionnaire for anxiety symptoms (Spitzer et al., 2006). Participants are asked to indicate how often they have been bothered by problems described in seven statements (e.g. “Feeling nervous, anxious or on edge”) over the previous 2 weeks. The same 4‐point Likert scale as the PHQ‐9 is used to sum responses for a total score ranging from 0 to 21, with higher scores corresponding to higher levels of anxiety symptoms.

#### Mini International Neuropsychiatric Interview v5.0.0 (MINI)

The MINI is a structured interview designed to assess mental health difficulties according to DSM‐IV and ICD‐10 diagnostic criteria (Sheehan et al., 1998). Specific MINI disorder modules were used to assess whether participants met the diagnostic criteria for any current low mood (major depression or dysthymia) or anxiety disorders (panic, agoraphobia, social anxiety or generalised anxiety).

### Statistical analysis

STATA Release 16 (StataCorp, 2019) was used for statistical analyses.

Multilevel mixed‐effects models with random intercepts of family group were used to compare continuous anxiety and depression scores across the different autism and ADHD groups. Mixed models were used to account for non‐independence in our sample (i.e. related twins; Malone et al., 2014). Anxiety and depression total scores were each set as the dependent variable in a separate multilevel model. Across all models, autism and ADHD grouping variables have been included as predictors, as well as their interaction term. Participant sex, age, cognitive ability, and parental SES have also been included as covariates based on pre‐registered analysis plans. Logistic regression models, adjusted for clustering of family group using variance‐covariance estimation were used to compare predictors related to participants meeting diagnostic thresholds on the MINI for low mood conditions or any anxiety condition.

Multiple testing was controlled using the false discovery rate method (Benjamini & Hochberg, 1995) across all predictors included in analysis models. Considering we had four models, PHQ‐9, GAD‐7, MINI low mood, and MINI anxiety, and three predictors (ADHD, autism, and their interaction) and four covariates (sex, age, cognitive ability, and parental SES) per model, 28 *p*‐values were ranked, and critical *q*‐values were calculated using a false discovery rate of 5%.

Missing data were handled with multiple imputation (MI) using chained equations (additional description in Supporting Information S1: S3). All analyses were carried out with complete case data only and with MI data. Results from analyses with MI data have been reported, and the two instances where significance patterns differed between MI and complete case analyses have been highlighted in the results.

Histograms were used to visually inspect distributions of non‐categorical variables (anxiety and depression scores, age, cognitive ability, and parental SES). Anxiety and depression scores were substantially non‐normally distributed and were transformed using a multivariate Box‐Cox procedure (Lindsey & Sheather, 2010; Velilla, 1993). Analyses were run with both transformed and non‐transformed scores, however no differences were observed in patterns of statistical significance. As such, analyses using non‐transformed variables have been reported for ease of interpretation.

Raincloud plots were produced using R and RStudio (R Core Team, 2021; RStudio Team, 2019) to visualise anxiety and depression total scores across groups. Raincloud plots combine split‐half violins, boxplots, and jittered raw data points (Allen et al., 2021), and provide a more open and detailed visualisation of the data than is possible with a single visualisation method.

## RESULTS

**FIGURE 1:**
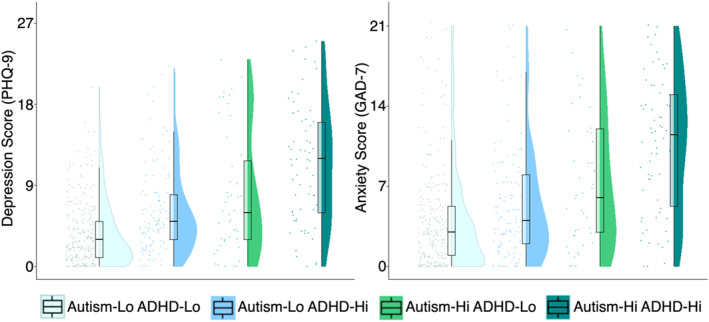
Raincloud plots showing the distribution of anxiety and depression scores across groups. Scores are separated by group; individuals with low autistic and ADHD traits (Autism‐Lo ADHD‐Lo), low autistic traits and high ADHD traits (Autism‐Lo ADHD‐Hi), high autistic traits and low ADHD traits (Autism‐Hi ADHD‐Lo), and those with high autistic and ADHD traits (Autism‐Hi ADHD‐Hi). For each group, jittered raw data are shown on the left, boxplots with median and interquartile range are shown in the middle, and density plots are shown on the right.

Descriptive statistics for each of the grouping and outcome measures are included in Table 1, divided by group and sex. Percentages of participants across groups meeting diagnostic thresholds on the MINI for any low mood diagnosis or anxiety condition are presented in Table 2. Table 3 reports coefficients or odds ratios and significance of all predictors and covariates in statistical models (multilevel mixed‐effects and logistic models for continuous questionnaire scores and diagnostic thresholds for mental health conditions respectively). Raincloud plots in Figure 1 display the medians and distributions of depression (PHQ‐9) and anxiety (GAD‐7) scores across groups.

**TABLE 1:** Descriptive statistics for each traits‐based group on all measures.

	Group															
	Autism‐Lo ADHD‐Lo				Autism‐Lo ADHD‐Hi				Autism‐Hi ADHD‐Lo				Autism‐Hi ADHD‐Hi			
	Male		Female		Male		Female		Male		Female		Male		Female	
Measure	*M*	*SD*	*M*	*SD*	*M*	*SD*	*M*	*SD*	*M*	*SD*	*M*	*SD*	*M*	*SD*	*M*	*SD*
ADOS‐2	1.19	0.47	1.07	0.28	1.3	0.58	1.11	0.37	3.54	3.06	3.29	2.52	3.45	3.13	2.9	2.49
SRS‐2 (raw score)	37.32	14.65	32.47	14.45	41.88	14.12	38.66	15.13	72.29	20.42	87.94	24.27	84	20.26	91.37	18.82
SRS‐2 (T‐score)	49	5.2	47.19	5.11	50.5	4.98	49.42	5.35	61.15	7.18	66.59	8.51	65.05	6.82	68.97	7.12
DIVA 2.0 (inattention)	1.1	1.32	1.21	1.47	3.27	2.5	3.47	2.19	1.75	1.56	2.77	1.72	4.82	2.35	5.83	2.07
DIVA 2.0 (hyperactivity)	2.01	1.68	1.71	1.55	3.09	2.34	2.95	2.07	2.29	1.15	2.88	1.73	3.82	1.88	4.5	2.05
DIVA 2.0 (areas)	3.11	2.58	2.91	2.52	6.36	4.1	6.42	3.61	4.04	1.99	5.65	2.64	8.64	3.08	10.33	3.33
BAARS‐IV	33.89	2.77	33.46	3.47	40.5	4.73	41.82	4.01	35.11	2.79	33.24	3.96	42.82	4.59	45.3	6.8
PHQ‐9	12.38	3.27	12.8	3.75	15.03	4.99	15.95	5.32	15.11	6.18	20.18	7.03	17.82	6.23	22.4	5.85
GAD‐7	2.58	3.81	4.65	4.62	5.02	5.44	6.41	5.07	5.32	5.57	10.36	4.58	8.52	5.72	13.29	5.3

*Note*: Table shows mean (*M*) and standard deviation (*SD*) for each of the grouping and outcome measures used, divided by trait group and sex. Scores for the DIVA 2.0 are broken down into subscales evaluating traits related to inattention and hyperactivity, as well as the number of areas in which participants reported experiencing problems.

**TABLE 2:** Number and percentage of participants across groups meeting diagnostic thresholds for any low mood or anxiety disorders using the MINI International Psychiatric Interview.

	Low mood				Anxiety			
	Depression or dysthymia				Panic disorder, agoraphobia, social anxiety, generalised anxiety disorder			
	Subthreshold		Meets Dx threshold		Subthreshold		Meets Dx threshold	
Trait grouping	*N*	%	*N*	%	*n*	%	*n*	%
Autism‐Lo ADHD‐Lo	284	95.95	12	4.05	245	82.77	51	17.23
Autism‐Lo ADHD‐Hi^a^	117	87.97	13	9.77	100	75.19	31	23.31
Autism‐Hi ADHD‐Lo	40	72.73	15	27.27	32	58.18	23	41.82
Autism‐Hi ADHD‐Hi	52	72.22	20	27.78	33	45.83	39	54.17

Abbreviation: Dx, Diagnostic.

^a^ 3 participants from this group (2.26%) are missing data on MINI depression and dysthymia modules, and 2 participants from this group (1.5%) are missing data on MINI anxiety modules.

**TABLE 3:** Prediction of continuous anxiety and depression scores and the likelihood of participants meeting thresholds for current low mood or anxiety disorders.

Mixed‐effects models	Predictor	Unstandardised coefficient	*p*‐value	*q*‐value	Sig. after correction
PHQ‐9	**Autism‐Hi**	**3.78**	**0.000**	**0.002**	*
	**ADHD‐Hi**	**2.70**	**0.000**	**0.004**	*
	Autism × ADHD	0.18	0.855	0.050	‐
	Age	−0.25	0.317	0.036	‐
	**Sex (male)**	**−1.50**	**0.001**	**0.014**	*
	**Cognitive ability**	**−0.58**	**0.015**	**0.021**	*^a^
	Parental SES	0.13	0.647	0.046	‐
GAD‐7	**Autism‐Hi**	**2.73**	**0.000**	**0.007**	*
	**ADHD‐Hi**	**1.85**	**0.001**	**0.018**	*
	Autism × ADHD	1.47	0.166	0.029	‐
	Age	0.11	0.641	0.045	‐
	**Sex (male)**	**−2.14**	**0.000**	**0.005**	*
	**Cognitive ability**	**−0.86**	**0.001**	**0.016**	*
	Parental SES	0.18	0.491	0.043	‐

Logistic models	Predictor	Odds ratio	*p*‐value	*q*‐value	Sig. after correction
MINI low mood	**Autism‐Hi**	**2.44**	**0.000**	**0.009**	*
	**ADHD‐Hi**	**1.14**	**0.006**	**0.020**	*^b^
	**Autism ×** **ADHD**	**−1.18**	**0.035**	**0.027**	‐
	Age	0.17	0.290	0.034	‐
	**Sex (male)**	**−0.75**	**0.021**	**0.023**	*
	Cognitive ability	−0.14	0.425	0.038	‐
	Parental SES	0.21	0.242	0.030	‐
MINI anxiety	**Autism‐Hi**	**1.55**	**0.000**	**0.013**	*
	**ADHD‐Hi**	**0.58**	**0.028**	**0.025**	‐
	Autism × ADHD	−0.17	0.725	0.048	‐
	Age	−0.09	0.484	0.041	‐
	**Sex (male)**	**−1.08**	**0.000**	**0.011**	*
	Cognitive ability	−0.14	0.281	0.032	‐
	Parental SES	0.10	0.480	0.039	‐

*Note*: Bold denotes a predictor significant at *p* < .05.

Abbreviations: ADHD‐Hi, high ADHD traits; Autism × ADHD, Autism and ADHD Group Interaction Term; Autism‐Hi, high autistic traits; *q*‐values, critical *q*‐values from multiple comparison correction procedure; Sig., significant.

^a^ Cognitive ability met significance threshold in models with multiply imputed data but did not meet threshold in complete case analysis.

^b^ High ADHD traits group met significance threshold in model with multiply imputed data but did not meet threshold in complete case analysis.

* Predictor remains statistically significant after correction for multiple comparisons.

### Depression and low mood

Summarised in Table 3, analysis of PHQ‐9 scores showed that Autism‐Hi participants reported significantly higher levels of depressive symptoms than the Autism‐Lo group, independent of ADHD grouping or covariates. Similarly, ADHD‐Hi participants reported significantly higher levels of depressive symptoms than ADHD‐Lo participants, independent of autism grouping. The ‘Autism × ADHD’ group interaction was not significant for depression symptoms, suggesting ADHD traits did not significantly exacerbate or reduce the effect of autistic traits on depression, or vice versa. Sex and cognitive ability were significant covariates in the model, with males reporting significantly lower levels of depression than females, as did those with higher levels of cognitive ability. Age and parental SES were not significantly related to participant‐reported depression symptoms.

Also shown in Table 3, the logistic regression model showed that the likelihood of meeting diagnostic criteria for a low mood disorder (depression or dysthymia) on the MINI was significantly higher among Autism‐Hi, compared to Autism‐Lo, participants, independent of ADHD grouping or other factors. Similarly, those in the ADHD‐Hi group were significantly more likely to meet these criteria than ADHD‐Lo participants. The ‘Autism × ADHD’ group interaction was significant at *p* < .05; however, this effect did not meet significance thresholds corrected for multiple testing. Males were significantly less likely to meet diagnostic criteria than females, but there were no significant effects of age, cognitive ability, or parental SES.

### Anxiety

Mixed‐effects analysis of GAD‐7 scores, represented in Table 3, showed that Autism‐Hi participants reported significantly higher levels of anxiety than the Autism‐Lo group, independent of ADHD grouping or covariates. Similarly, ADHD‐Hi participants reported significantly higher anxiety symptoms than ADHD‐Lo participants, independent of autism grouping. The ‘Autism × ADHD’ group interaction was not significant. Males reported significantly lower anxiety levels than females, as did those with higher levels of cognitive ability. Age and parental SES were not significantly related to participant reported anxiety symptoms.

Logistic models also summarised in Table 3 showed that the likelihood of meeting diagnostic criteria for an anxiety disorder (panic, agoraphobia, social anxiety, or generalised anxiety disorder) on the MINI was significantly higher among Autism‐Hi compared to Autism‐Lo participants independent of ADHD grouping or other factors. Similarly, those in the ADHD‐Hi group were significantly more likely to meet these criteria than ADHD‐Lo participants. The ‘Autism × ADHD’ group interaction was not significant, suggesting ADHD traits did not significantly exacerbate or reduce the effect of autistic traits on anxiety, or vice versa. Males were significantly less likely to meet diagnostic criteria than females, but there were no significant effects of age, cognitive ability, or parental SES.

### Additional analyses

Additional exploratory analyses examining potential interactions between autism/ADHD and sex have been reported in Supporting Information S1: S6. There were no significant interaction effects of ADHD with sex for any of the mental health outcome measures. However, there were significant interaction effects between autistic traits and sex for depression and anxiety scores from the PHQ‐9 and GAD‐7. In both cases, it appeared that the association between high autistic traits and elevated self‐reported mental health symptoms was more pronounced in females compared to males. The interaction effect between autism and sex was non‐significant for predicting the likelihood of meeting criteria for any low mood or anxiety disorders as measured by the MINI.

Finally, as evidenced by the raincloud plots for each group (Figure 1), scores in the ‘Autism‐Hi ADHD‐Hi’ group exhibited considerably greater range and distribution than those of the other groups.

## DISCUSSION

Consistent with our hypotheses, we found that high levels of both autistic traits and ADHD traits significantly and independently associated with higher self‐reported symptoms of depression and anxiety, as well as a greater likelihood of meeting diagnostic criteria for low mood or anxiety disorders, independent of age, sex, cognitive ability, and parental SES. However, the interaction between high autistic and high ADHD traits was not significantly associated with depression and anxiety. Consequently, their co‐occurrence was significantly associated with a heightened risk of mental health problems but appears to reflect a plateau, wherein the overall strength of the association is increased, though not in a synergistic manner. The visualised data in Figure 1 nonetheless underscore the wide range and dispersion of depression and anxiety scores in the group with high levels of both autistic and ADHD traits.

We found that females were more likely to report higher levels of depression and anxiety and had a higher likelihood of meeting diagnostic criteria, after controlling for other variables. This is unsurprising given the established sex differences in rates of internalising conditions in the general population (McManus et al., 2016), as well as in autistic (Sedgewick et al., 2020; Uljarević et al., 2021), and ADHD (Chen et al., 2018) adult samples. Interestingly, our results suggest that sex might interact with levels of autistic, though not ADHD, traits (Supporting Information S1: S6). The interaction was such that the positive relationship between autistic traits and internalising symptoms was stronger for females compared to males. This finding implies that women with high autistic traits may have an especially heightened risk for experiencing internalising problems. However, it should be noted that these findings are based on biological sex and not experienced gender identity, which might exhibit a differential relationship with mental health. Similarly, more recent indicators of the young adults' SES, as opposed to the measure of parental SES in childhood, might also be relevant and may be more likely to show associations with current mental health. Evidence from three large longitudinal cohorts with health record linkage suggests that low SES in adulthood is associated with poor mental health outcomes (Kivimäki et al., 2020).

Our findings differ from some larger‐scale health record studies examining diagnosed autism and ADHD. For instance, Solberg et al. (2019) found no significant differences in rates of depression between adults with ADHD and adults with both autism and ADHD, although prevalence ratios were numerically highest among the combined group. Furthermore, Chen et al. (2015) found no differences in rates of anxiety among those with ADHD compared to those with both autism and ADHD. While we found no evidence for a multiplicative interaction between trait groups, our findings nonetheless indicate that the co‐occurrence of ADHD and autism is predictive of increased risk for internalising symptoms overall.

In line with previous work, these findings highlight a number of important implications. One central implication of particular clinical relevance may be the need for specialised interventions and care, with specific barriers to accessing support emerging as a prominent theme for adults with neurodevelopmental differences and co‐occurring mental health conditions (Camm‐Crosbie et al., 2019). Not only is there an established lack of evidence‐based interventions for mental health challenges adapted to individuals with neurodivergent traits (Lipinski et al., 2022; Pantazakos, 2023), many care providers report feeling ill‐equipped to offer adequate support, even when well‐informed about such traits and characteristic presentations (Adams & Young, 2021; Unigwe et al., 2017; Zerbo et al., 2015). Further complicating this, both communication challenges and difficulties in identifying pathological changes in mental health, for example, due to the well‐documented alexithymia associated with autism, may contribute to this issue (Bird et al., 2010; Crane et al., 2019). The very traits that might predispose an individual to mental health difficulties can thus impact their ability to recognise the problems, effectively seek help, and benefit from the help provided (Camm‐Crosbie et al., 2019; Lugo‐Marín et al., 2019).

As evidenced by our results, as well as many existing studies, sex differences can also strongly influence expression and associated outcomes of both ADHD and autistic traits (Groß‐Lesch et al., 2016; Ottosen et al., 2019). Females appear to have a particular vulnerability to these associations, especially those exhibiting high levels of autistic traits, which may be amplified in young adulthood by a comparatively reduced tendency to ‘grow out’ of symptoms (Owens et al., 2009). The well‐documented phenomenon of masking likely contributes to these observed sex differences, as well as to a wider consideration of diagnostic overshadowing (Chandrasekhar & Sikich, 2015; Hollocks et al., 2019; Martin, 2024). While compensatory strategies may convey an impression of coping, they may nevertheless obscure the true picture and further lead to underestimation of the severity of mental health difficulties in question (Camm‐Crosbie et al., 2019; Kenny et al., 2016).

These considerations could inform the development of more specialised care in this context. Practical applications of one such approach could include support with emotional literacy prior to psychological interventions, which might strengthen rapport building, optimise the efficacy of, and reduce strain on already‐limited available resources (Anderberg et al., 2017; Camm‐Crosbie et al., 2019). Better‐informed interventions may also target executive function demands in early adulthood to mitigate their effects on mental health in individuals with high autistic or ADHD traits (Aydin et al., 2022; Lawson et al., 2015). Accommodations could also be made for differential social experiences and emotional regulation abilities, which appear to powerfully mediate relationships between these traits and internalising symptoms (Camus et al., 2024; Stice & Lavner, 2019). In this sense, broader psychoeducation not only for practitioners but also for peers of adults in this cohort could confer a profound positive impact (Gillespie‐Lynch et al., 2015; Riglin et al., 2023; Smith et al., 2012). In light of high levels of social stigma attached to autism, ADHD, *and* mental health conditions, stronger individualised supports would appear essential for individuals ‘doubly disadvantaged’ in this way (Crane et al., 2019).

Findings from the present study, however, cannot speak to the effectiveness of psychological interventions, and must be interpreted with consideration for several limitations. As previously mentioned, the lack of information regarding gender identity as distinct from biological sex is one limitation of note, as is the measure of SES included here which may hold less of a bearing on participants' SES at the time of testing. Additionally, our results on cognitive ability and mental health are consistent with previous findings of ambiguous effects; though higher cognitive ability may be generally associated with stronger global adaptive functioning, this appears less pronounced among those with neurodevelopmental differences (Åsberg Johnels et al., 2021). The interpretation of this relationship in this sample is limited by the exclusion of individuals with an estimated IQ below 70. While a community‐based sample offers distinct advantages, and may shed greater light on associations that could be eclipsed by missed or misdiagnoses in clinical settings, especially with respect to diagnostic overshadowing (Jopp & Keys, 2001), the present sample is still a selected sample (i.e. based on adolescent neurodevelopmental traits) and so is likely to present with its own idiosyncratic biases. Moreover, though advantageous for several reasons, the use of both self‐ and other‐report measures in this context inherently introduces some degree of additional variability. This holds particular relevance for self‐report measures such as the SRS‐2 which has demonstrated some difficulty in disentangling autistic traits from some anxiety symptoms (South et al., 2017), despite reasonably strong specificity overall (Moody et al., 2017). As Uljarević et al. (2016) argue, prevalent anxiety among individuals with autism may represent an inevitable consequence of coping with normative demands and societal expectations. Though this suggests a need for caution in interpreting the current findings, it also reflects a recurring issue in psychological research in teasing apart complex and interrelated constructs, further complicated by the transdiagnostic nature of psychopathology more generally, but especially in this group (Stanton et al., 2021).

Several avenues for future research have thus been highlighted. Future investigations may seek to directly compare data from several sources, such as self‐report, relative‐report, and behavioural assessments. In addition, further quantitative studies could provide an empirical review of psychological supports and interventions tailored for individuals with high autistic, ADHD, or both traits, including analysis of any emerging sex differences. Along this line, further work may explore the protective factors at play which may mitigate the impact of these traits on young adult mental health, adopting a lifespan perspective and potentially focusing on the social aspects involved.

In essence, our findings align with existing work that indicates young adult traits of neurodivergence may be significantly complicated and even exacerbated by concomitant mental health challenges (Pearl et al., 2017). Through continued elaboration, the multifaceted dimensional associations between the conditions studied here may even challenge future classifications in clinical practice (Lundström et al., 2011). Taken together, findings to date suggest routine screening for these traits in general mental health services may identify previously undetected patterns of behaviour, allowing patients to further benefit from specialised support (Adamis et al., 2018). While promoting psychoeducation is crucial, given that autistic adults (Camm‐Crosbie et al., 2019; Mason et al., 2019) and adults with ADHD (Matheson et al., 2013) frequently report barriers accessing care, improving awareness alone may be inadequate without addressing inequalities in access. Accessibility barriers represent an issue that is not exclusive to individuals with high levels of autistic or ADHD traits, but rather endemic to mental health services, yet may have discrete consequences for those in this cohort navigating systems designed around neurotypical norms (Crane et al., 2019). Crucially, however, as emphasised by Camm‐Crosbie et al. (2019), adaptation of services and care is not only possible, but highly beneficial.

## AUTHOR CONTRIBUTIONS

**Simone Capp**: Data curation; formal analysis; methodology; project administration; writing – original draft. **Aoibhe De Burca**: Formal analysis; writing – review and editing. **Ümit Aydin**: Data curation; writing – review and editing. **Jessica Agnew‐Blais**: Formal analysis; investigation; methodology; supervision; visualization; writing – review and editing. **Alexandra Lautarescu**: Project administration; writing – review and editing**. Angelica Ronald**: Supervision; writing – review and editing. **Francesca Happé**: Formal analysis; investigation; supervision; writing – review and editing. **Gráinne McLoughlin**: Conceptualization; data curation; formal analysis; funding acquisition; investigation; methodology; project administration; resources; supervision; validation; visualization; writing – review and editing.

## CONFLICT OF INTEREST STATEMENT

The authors declare no conflicts of interest.

## ETHICAL CONSIDERATIONS

The King's College London Psychiatry, Nursing and Midwifery Research Ethics Subcommittee granted ethical approval for the study (RESCMR‐16/17–2673) on the 2nd of June 2016. Written informed consent was obtained from all participants.

## Supporting information

Supporting Information S1

## Data Availability

The data that support the findings of this study are available from the corresponding author upon reasonable request.
