# Supplementary material for: Depression and anxiety are increased in autism and ADHD: Evidence from a young adult community‐based sample
Source: JCPP Adv. 2025 Mar 26;5(4):e70003. doi: 10.1002/jcv2.70003 (PMC12698284; doi:10.1002/jcv2.70003)
Supplement: Supplementary file 1 — Supporting Information S1 [file JCV2-5-e70003-s001.docx]

# **Supporting Information**

**S1. IDEAS Recruitment Pathways**

Participants were selected and invited to participate in the Individual Differences in EEG in young Adults Study (IDEAS) based on their previous research participation in the Twins Early Development Study (TEDS). The four recruitment paths are described below:

***Path 1-*** The Social Relationships (SR) Study is an established subsample from TEDS focusing on families where one or both twins met diagnostic criteria for an autism spectrum condition or had high levels of autistic traits in adolescence (more details in Colvert et al., 2015). To date, there have been three phases to the SR Study. Phase 1 collected data when participants were adolescents.

A total of 290 participants from the SR Study Phase 1 sample were selected for invitation to IDEAS (126 autism group, 50 low-traits co-twins, 114 comparison twins). Out of those selected, 92 were contacted successfully and agreed to participate in IDEAS (26 autism group, 14 co-twins, 52 comparison twins). Only those estimated to have an IQ of 70 or greater in adolescence were invited to participate in IDEAS (82 SR Study participants were not selected for participation in IDEAS on this basis).

***Path 2-*** The Neurophysiological Study of Activity and Attention in Twins Study (NEAAT) is a subsample of male TEDS twins tested at age 14, focusing on those with high levels of ADHD traits, plus a comparison group of those with low ADHD traits (more details in Tye et al., 2012). A total of 130 participants from NEAAT were selected for invitation to IDEAS (49 ADHD, 8 low-traits co-twins, 73 comparison twins). Of those selected, 62 were contacted successfully and agreed to participate in IDEAS (21 ADHD, 3 low-traits co-twins, 38 comparison twins).

***Path 3-*** The SR Study completed the third phase of data collection when the twins were aged 20-25. In SR Study phase 3, additional participants were recruited from TEDS who reported high levels of autistic traits or an autism diagnosis since the SR Study phase 1. Of the participants new to the SR Study 3 Phase 3 (who had not been part of SR Phase 1 or NEAAT), 64 were invited to participate in IDEAS. Of those invited, 48 went on to participate in IDEAS. These participants either had a formal autism diagnosis (*n =* 15) or were considered by the research team to display high levels of autistic traits (*n =* 33) based on various records held by TEDS.

***Path 4-*** The remaining participants invited to participate in IDEAS from TEDS had not previously participated in the SR Study or NEAAT. This included additional female and opposite-sex twin pairs with high ADHD traits and additional comparison twins with low levels of autistic and ADHD traits. A total of 660 participants were identified from the main TEDS cohort and invited to participate in IDEAS; 354 were successfully contacted and took part in the study.

**S2. Measures**

***Autism Diagnostic Observation Schedule (ADOS-2)***

Module 4 of the ADOS-2 (suitable for fluent speech), which includes a range of activities and interview questions, was used to assess whether participants met diagnostic criteria for autism spectrum conditions. For this module, researchers rate behaviours observed within the assessment against 32 criteria relating to typical autistic characteristics and behaviours. Possible scores for each criterion range from 0 to 2 or 3, with higher scores representing more definite and pronounced autistic behaviours. Calibrated Severity Scores (CSS) were calculated as standard, ranging from 1-10, and scores of 4 or higher were taken to indicate behaviours and characteristics consistent with an autism spectrum diagnosis.

***Social Responsiveness Scale (SRS-2)***

The SRS-2 adult self-report questionnaire includes 65 items to assess social and behavioural autism characteristics, e.g. "I am awkward in turn taking interactions with others - for example, I have a hard time keeping up with the give-and-take of a conversation", “I am much more uncomfortable in social situations than when I am by myself”. Items are rated using a 4-point Likert scale (0 = Not true, 1 = Sometimes true, 2 = Often true, 3 = Almost always true). Several items in this measure have reversed scoring, conveying social proficiencies more typical of non-autistic populations. Raw sum scores are calculated from the numeric scores assigned to each item and range from 0-195. Raw scores of 68 (or standardised T-scores of 60) or higher are considered to identify significant autism-related social and behavioural difficulties, with extensive validity testing supporting its use in conjunction with other behavioural assessments (Bruni, 2014).

***Diagnostic Interview for ADHD in Adults (DIVA 2.0)***

During the DIVA 2.0, participants are asked about their experience of ADHD symptoms, both currently and during childhood (e.g., "Do you often find it difficult to sustain your attention on tasks?"). The interview includes nine items relating to symptoms of inattention and nine items for hyperactivity/impulsivity symptoms. Participants are also asked to report first onset of symptoms and whether their reported symptoms have caused problems in different life domains (work/education, relationship or family, social contacts, free time/hobby, self-confidence/self-image). The measure has been shown to reliably discriminate adult ADHD with strong validity (Pettersson et al., 2018; Ramos-Quiroga et al., 2019).

***Barkley Adult ADHD Rating Scale-IV (BAARS-IV)***

The BAARS-IV self-report questionnaire includes 27 items assessing characteristics associated with ADHD (e.g. “Difficulty sustaining my attention in tasks or fun activities”, “[I] Have difficulty organising tasks and activities”). Participants rate how often they experienced each item over the previous six months, using a 4-point Likert scale (1 = Never or rarely, 2 = Sometimes, 3 = Often, 4 = Very often). Sum scores were produced from all items across three domains (inattention, hyperactivity, and impulsivity), with a possible range from 18-72, where higher scores indicate greater ADHD-related traits and behaviours. A cut-off score of 39 or above was used to identify those with mild-marked ADHD symptoms, consistent with official BAARS-IV guidelines (Barkley, 2011). This measure has been well validated for use with this age group (Caroline et al., 2024; Saleh et al., 2018).

***Patient Health Questionnaire (PHQ-9)***

The PHQ-9 is a nine-item questionnaire commonly used to screen for depression symptoms. For each statement, e.g. "Little interest or pleasure in doing things", “Feeling down, depressed, or hopeless”, participants are asked to rate how often this has applied to them over the previous two weeks using a 4-point Likert scale (0 = Not at all, 1 = Several days, 2 = More than half the days, 3 = Nearly every day). Item scores are summed for a continuous total score which ranges from 0-27, with higher scores indicating greater levels of depressive symptoms. The measure has been validated for detection of both major depressive disorder and subthreshold depressive symptoms in the general population (Martin et al., 2006).

***Generalised Anxiety Disorder Assessment (GAD-7)***

Designed for complementary use with the PHQ-9, the GAD-7 assesses anxiety symptoms by asking respondents how often they have been bothered over the previous two weeks by problems described in seven statements (e.g. "Feeling nervous, anxious or on edge", “Not being able to stop or control worrying”). Participants respond using the same 4-point Likert scale as used with the PHQ-9. Similarly, a total sum score is derived, ranging from 0-21, where higher scores indicate greater levels of anxiety symptoms. Evidence supports the use of the GAD-7 as a measure of anxiety in the general population (Löwe et al., 2008).

# ***Mini International Neuropsychiatric Interview (MINI)***

In the current study, MINI modules assessing current major depression, dysthymia, panic disorder, agoraphobia, social anxiety disorder, obsessive-compulsive disorder, and generalised anxiety disorder (GAD) were used. Using a structured, standard hierarchy of interview questions, the interviewer determines whether a participant would meet current diagnostic criteria for each disorder assessed. Results from this interview were used to classify whether participants met the diagnostic criteria for any current low mood or anxiety disorders. The MINI has demonstrated strong reliability and validity in facilitating these diagnoses in a short time frame (Lecrubier et al., 1997).

***Cognitive ability***

Versions of the Raven's Standard Progressive Matrices (Raven, 1960) and the Mill Hill Vocabulary Scale (Raven, 1962) were included in the online survey completed by IDEAS participants. Scores from these tests have been used and combined to provide a measure of general cognitive ability for IDEAS.

A 30-item version of Raven's Standard Progressive Matrices was used to assess non-verbal cognitive ability. For each item in the test, participants were presented with a logical picture matrix. Participants were also shown eight possible solutions to complete the matrix and asked to identify which one correctly completes the sequence. Correct responses were given a score of 1, and all incorrect responses a score of 0. As such, possible scores ranged from 0-30.

A 33-item version of the Mill Hill Vocabulary Scale was used as a complementary assessment of verbal cognitive ability. For each item in the test, participants were presented with a target word and six potential 'solution words'. Participants were asked to choose the word with the closest meaning to the target word. Participants were given 10 seconds to respond to each item to reduce the chances of cheating. As before, the correct response was given a score of 1, and all other options scored 0.

Scores from both these tests were standardised and combined to produce a cognitive ability composite score. The measures have previously been used online and used to create cognitive ability composite scores (see Haworth et al., 2007; von Stumm & Plomin, 2015).

***Parental socioeconomic status***

An early life parental socioeconomic status (SES) composite was created from information on parent qualifications and employment, and maternal age at birth of first child. These measures were collected by TEDS when participants were 18 months of age. This 18-month parental SES composite has been used and described previously (Hanscombe et al., 2012; von Stumm & Plomin, 2015).

**S3. Missing Data and Multiple Imputation**

Between 0-13% of participants were missing data on key measures (see table below).

| **Table S3**  *Number and percentages of participants missing data for key measures (from the total sample of 556)* | | |
| --- | --- | --- |
| **Measure** | ***n*** | **%** |
| MINI | 1 | <1% |
| PHQ-9 | 67 | 12% |
| GAD-7 | 73 | 13% |
| ADOS-2 | 9 | 2% |
| SRS-2 | 73 | 13% |
| DIVA 2.0 | 0 | 0% |
| BAARS-IV | 66 | 12% |

Logistic regression, Spearman correlations and t-tests were used to identify variables related to missingness or associated with scores for key variables. Data were assumed to be missing at random as several variables were identified, which were related to data missingness and scores from incomplete variables.

Multiple imputation via chained equations (Royston & White, 2011) has been used to handle missing data for analyses. Twenty multiply imputed sets were created and pooled for analyses. The following variables were included in the multiple imputation specification based on their relationships to data missingness and key variable scores:

Social Relationships Study Phase 1 participation, ADOS-2 module 4 algorithm total scores, DIVA 2.0 total symptoms, age (at DIVA 2.0 completion and online questionnaire completion), general cognitive ability score, sex, MINI conditions endorsed (major depression, dysthymia, panic disorder, agoraphobia, social anxiety, obsessive compulsive disorder and generalised anxiety), PHQ-9 sum scores, GAD-7 sum scores, SRS-2 sum score, BAARS-IV total score, and World Health Organisation Quality of Life-BREF (WHOQOL-BREF) domain scores.

The WHOQOL-BREF has not been used in the current study other than for multiple imputation and is described below.

The WHOQOL-BREF is a commonly used 26‑item measure of quality of life that asks participants to consider aspects of their lives over the past two weeks (The WHOQOL Group, 1998; World Health Organization, 1996). Each item has a 5-point Likert response scale which varies according to specific question phrasing. The WHOQOL-BREF has two broad summary items and 24 items divided across four subdomains (physical, psychological, social, environmental). For each domain, possible scores range from 4-20, with higher scores indicating higher quality of life in that area (World Health Organization, 1996).

**S4. Traits-based Group Frequencies**

*Frequency table showing the number of participants assigned to each group on the basis of high/low autistic or ADHD traits*

|  |  | **Number and proportion** | | | | |  | |  | | | | |
| --- | --- | --- | --- | --- | --- | --- | --- | --- | --- | --- | --- | --- | --- |
|  |  | Male | | | Female | | | | Total | | | | |
| **Trait grouping** | | | *n* | *%* | | *n* | | *%* | | *n* | *%* | | |
| Autism-Lo ADHD-Lo | | | 117 | 43.82 | | 179 | | 61.94 | | 296 | | 53.24 |  |
| Autism-Lo ADHD-Hi | | | 75 | 28.09 | | 58 | | 20.07 | | 133 | | 23.92 |  |
| Autism-Hi ADHD-Lo | | | 36 | 13.48 | | 19 | | 6.57 | | 55 | | 9.89 |  |
| Autism-Hi ADHD-Hi | | | 39 | 14.61 | | 33 | | 11.42 | | 72 | | 12.95 |  |
| **Total** *(n)* | | | 267 |  | | 289 | |  | | 556 | |  |  |

*Note.* Group composition percentages for ‘Male’ and ‘Female’ columns represent the percentage of male and female participants in the overall sample respectively.

**S5. Group Inclusion Criteria**

**Table S5a**

*Breakdown of number and proportion of participants per group meeting either/both of the criteria for inclusion in the ‘Autism-Hi’ group*

|  |  | **Group inclusion criterion** | | | | | | | | | | |
| --- | --- | --- | --- | --- | --- | --- | --- | --- | --- | --- | --- | --- |
|  |  | ADOS-2 | | | SRS-2 | | | Both | | | | |
| **Trait grouping** | | | *n* | *%* | | *n* | *%* | | *n* | *%* | | |
| High autistic traits | | | 26 | 20.47 | | 80 | 62.99 | | 21 | | 16.54 |  |

*Note.* Table represents number of individuals meeting criteria for inclusion in ‘Autism-Hi’ group. Percentages represent the proportion of individuals in either of these groups (*n* = 127) meeting specific criteria.

**Table S5b**

*Breakdown of number and proportion of participants per group meeting either/both of the criteria for inclusion in the ‘ADHD-Hi’ group*

|  |  | **Group inclusion criterion** | | | | | | | | | | |
| --- | --- | --- | --- | --- | --- | --- | --- | --- | --- | --- | --- | --- |
|  |  | DIVA 2.0 | | | BAARS-IV | | | Both | | | | |
| **Trait grouping** | | | *n* | *%* | | *n* | *%* | | *n* | *%* | | |
| High ADHD traits | | | 42 | 20.49 | | 105 | 51.23 | | 58 | | 28.29 |  |

*Note.* Table represents number of individuals meeting criteria for inclusion in ‘ADHD-Hi’ group. Percentages represent the proportion of individuals in either of these groups (*n* = 205) meeting specific criteria.

**S6. Exploratory Analyses Examining Autistic Traits, ADHD Traits and Sex Interactions**

Exploratory analyses were performed to examine whether there were interactions between autistic traits, ADHD traits, and sex in relation to continuous anxiety and depression ratings and the likelihood of meeting diagnostic thresholds for any current low mood or anxiety disorders. Analysis models replicated those presented in the main manuscript. Multilevel mixed-effects models (with a random intercept of family group) were performed predicting depression (PHQ-9) and anxiety (GAD-7) scores separately. Logistic regressions adjusted for clustering (of family group) were used to compare factors related to the likelihood of participants meeting diagnostic thresholds on the MINI for low mood conditions or any anxiety condition. Similarly, participant sex, age, cognitive ability, and SES have also been included as covariates. Analyses have been performed using multiply imputed data and full case data (as well as with transformed PHQ-9 and GAD-7 variables) and compared for consistency.

Analyses were initially performed considering the complete three-way interaction between variables (i.e. autistic traits group x ADHD traits group x sex plus all other interaction combinations). For all dependent variables, the interaction between autistic traits group and ADHD traits group was non-significant at *p* < .05. In addition, the three-way interaction term was also a non-significant predictor in all models. We therefore ran additional simplified models using two two-way interactions (autistic traits group x sex *and* ADHD traits group x sex) for ease of interpretation. Multiple testing was controlled using the false discovery rate method (Benjamini & Hochberg, 1995) across all predictors included in analysis models. Across all models, for 32 predictors (8 each in four models), p-values were ranked, and critical q-values were calculated using a false discovery rate of 5%. Coefficients and significance of predictors in models predicting depression (PHQ-9) and anxiety (GAD-7) scores and the likelihood of participants meeting diagnostic thresholds on the MINI for low mood conditions or any anxiety condition are presented in Table S6 below.

| **Table S6**  *Prediction of continuous anxiety and depression scores and the likelihood of participants meeting thresholds for current low mood or anxiety disorders: consideration of autistic traits and ADHD traits and interactions with sex* | | | | | |
| --- | --- | --- | --- | --- | --- |
| Mixed-effects models | Predictor | Unstandardised coefficient | p-value | q-value | Sig. after correction |
| PHQ-9 | **Autism-Hi** | **6.24** | **0.000** | **0.003** | ***** |
|  | **ADHD-Hi** | **2.77** | **0.000** | **0.006** | ***** |
|  | Sex (male) | -0.42 | 0.461 | 0.039 |  |
|  | **Autism-Hi x sex** | **-4.17** | **0.000** | **0.005** | ***** |
|  | ADHD-Hi x sex | -0.32 | 0.708 | 0.045 | - |
|  | Age | -0.21 | 0.371 | 0.033 | - |
|  | **Cognitive ability** | **-0.58** | **0.015** | **0.017** | ***a** |
|  | Parental SES | 0.06 | 0.816 | 0.048 | - |
| GAD-7 | **Autism-Hi** | **5.32** | **0.000** | **0.008** | ***** |
|  | **ADHD-Hi** | **1.60** | **0.018** | **0.019** | ***a** |
|  | **Sex (male)** | **-1.83** | **0.002** | **0.014** | ***** |
|  | **Autism-Hi x sex** | **-3.16** | **0.004** | **0.016** | ***b** |
|  | ADHD-Hi x sex | 0.94 | 0.312 | 0.028 | - |
|  | Age | 0.13 | 0.596 | 0.044 | - |
|  | **Cognitive ability** | **-0.86** | **0.001** | **0.011** | ***** |
|  | Parental SES | 0.16 | 0.560 | 0.042 | - |
| Logistic models | Predictor | Odds ratio | p-value | q-value | Sig. after correction |
| MINI Low Mood | **Autism-Hi** | **1.82** | **0.000** | **0.009** | ***** |
|  | **ADHD-Hi** | **0.97** | **0.028** | **0.020** | **-** |
|  | Sex (male) | -0.07 | 0.884 | 0.050 | - |
|  | Autism-Hi x sex | -0.16 | 0.800 | 0.047 | - |
|  | ADHD-Hi x sex | -1.00 | 0.141 | 0.022 | - |
|  | Age | 0.15 | 0.346 | 0.031 | - |
|  | Cognitive ability | -0.15 | 0.402 | 0.034 | - |
|  | SES | 0.21 | 0.261 | 0.023 | - |
| MINI Anxiety | **Autism-Hi** | **1.66** | **0.000** | **0.002** | ***** |
|  | ADHD-Hi | 0.31 | 0.285 | 0.027 | - |
|  | **Sex (male)** | **-1.17** | **0.002** | **0.013** | ***** |
|  | Autism-Hi x sex | -0.34 | 0.486 | 0.041 | - |
|  | ADHD-Hi x sex | 0.45 | 0.332 | 0.030 | - |
|  | Age | -0.09 | 0.458 | 0.038 | - |
|  | Cognitive ability | -0.14 | 0.275 | 0.025 | - |
|  | Parental SES | 0.11 | 0.457 | 0.036 | - |
| *Note.* Bold denotes predictor significant at *p* < .05.  * Predictor significant after correction for multiple comparisons. ^a^ Predictor met significance threshold in models with multiply imputed data but did not meet threshold in complete case analysis. ^b^ The autistic traits by sex interaction was significant in models with non-transformed data, but not in models with Box-Cox transformed GAD-7 variable. Autism-Hi = high autistic traits. ADHD-Hi = high ADHD traits. Sig. = significant. q-value = critical q-values from multiple correction procedure. | | | | | |

**Depression and Low Mood**

Analysis of PHQ-9 scores showed that Autism-Hi participants reported significantly higher depression than the Autism-Lo group, independent of ADHD grouping and other factors. Similarly, ADHD-Hi participants reported significantly higher depression symptoms than ADHD-Lo participants, independent of autism group. Sex was not identified as a significant predictor of PHQ-9 depression scores, nor was the sex by ADHD group interaction term. However, the sex by autistic traits group interaction was a significant predictor of depression scores. This interaction was such that the magnitude of the relationship between high autistic traits and depression scores was lower among males than females. This interaction is visualised in Figure S6a. Higher cognitive ability was significantly associated with lower depression scores among participants. Age and SES were not significant predictors.

**Figure S6a**

*Prediction of Continuous Depression Scores*


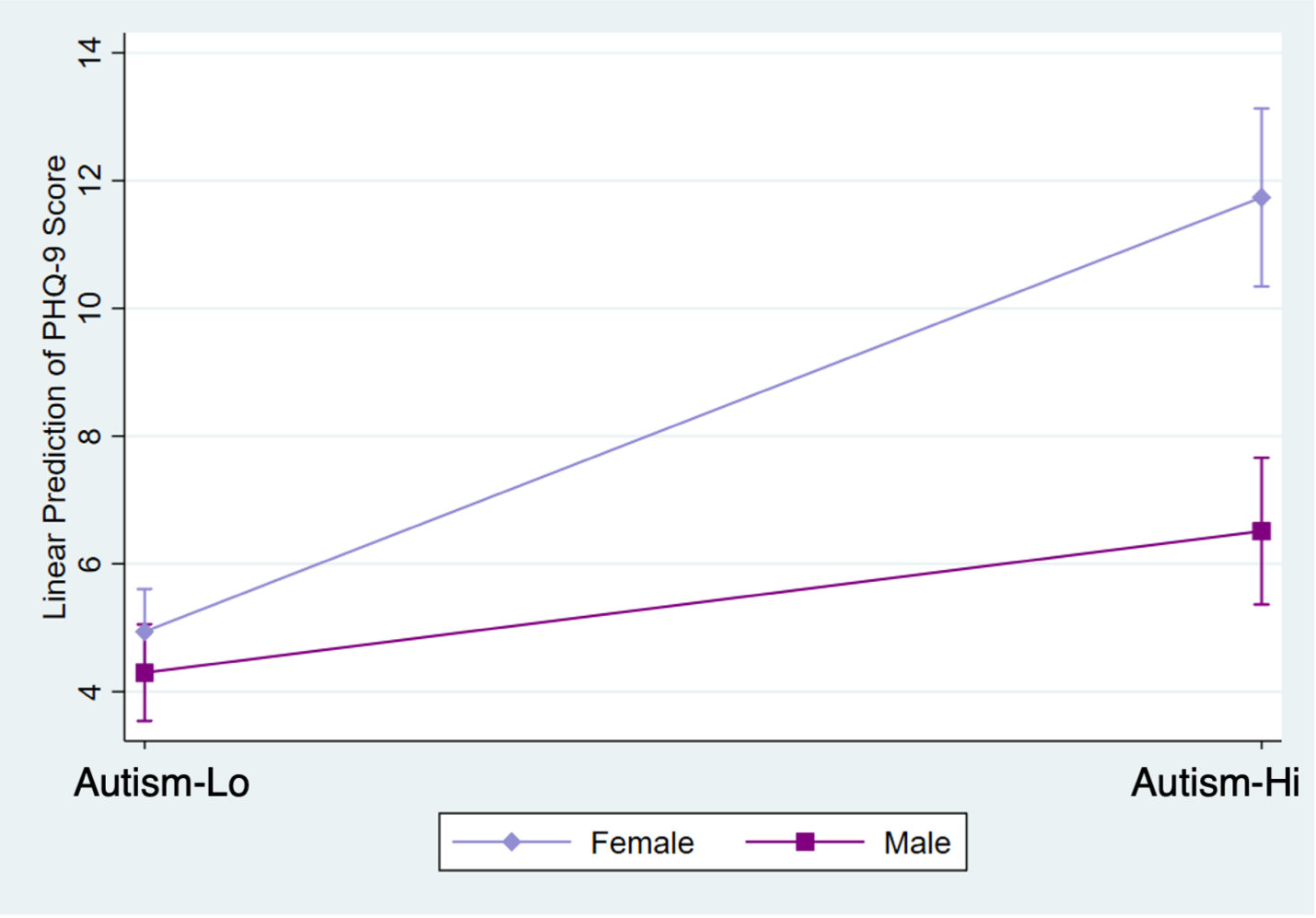


*Note.* Plot shows the linear prediction of PHQ-9 depression scores in those with low (Autism-Lo) and high (Autism-Hi) autistic traits (independent of ADHD traits).

Logistic models showed that the likelihood of meeting diagnostic criteria for a low mood disorder (depression or dysthymia) using the MINI was significantly higher among Autism-Hi, compared to Autism-Lo, participants, independent of ADHD grouping and other factors. The effect of ADHD group was significant at *p* < 0.05 but did not meet corrected significance levels. No other significant predictors were identified relating to the likelihood of meeting criteria for a low mood disorder using the MINI, this included sex, both sex by group interactions, age and cognitive ability.

**Anxiety**

Analysis of GAD-7 scores showed that Autism-Hi participants reported significantly higher anxiety than the Autism-Lo group, independent of ADHD grouping and other factors. Similarly, ADHD-Hi participants reported significantly higher anxiety symptoms than ADHD-Lo participants, independent of autism group. Males reported significantly lower levels of anxiety than females. The sex by autistic traits group interaction was a significant predictor of anxiety scores. This interaction was such that males with high autistic traits had lower anxiety scores than would be expected based on the independent additive effect of autistic traits group and sex. This interaction is visualised in Figure S6b. The sex by ADHD group interaction term was non-significant. Those with higher cognitive ability reported significantly lower anxiety, but age and SES were not significant predictors.

**Figure S6b**

*Prediction of Continuous Anxiety Scores*


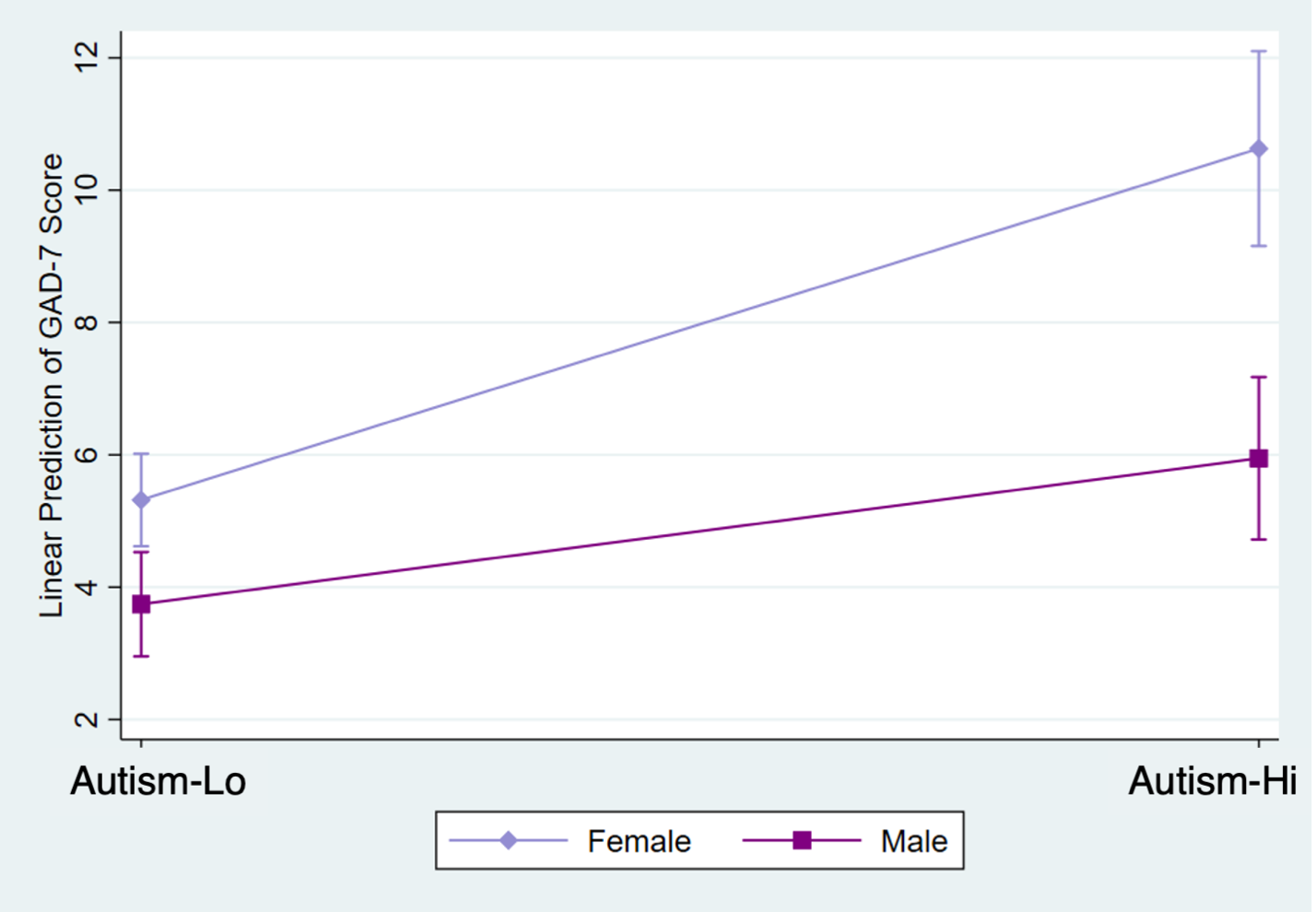


*Note.* Plot shows the linear prediction of GAD-7 anxiety scores in those with low (Autism-Lo) and high (Autism-Hi) autistic traits (independent of ADHD traits).

Logistic models showed that the likelihood of meeting diagnostic criteria for an anxiety disorder (panic disorder, agoraphobia, social anxiety or generalised anxiety) using the MINI was significantly higher among Autism-Hi, compared to Autism-Lo, participants, independent of ADHD grouping and other factors. ADHD grouping was not a significant predictor in this model. Males were significantly less likely to meet diagnostic criteria than females, but there were no significant effects of either interaction terms, age, cognitive ability, or SES.

**Supplementary References**

Bruni, T. P. (2014). Test Review: Social Responsiveness Scale–Second Edition (SRS-2). *Journal of Psychoeducational Assessment*, *32*(4), 365–369. https://doi.org/10.1177/0734282913517525

Caroline, S. S., Sudhir, P. M., Mehta, U. M., Kandasamy, A., Thennarasu, K., & Benegal, V. (2024). Assessing Adult ADHD: An Updated Review of Rating Scales for Adult Attention Deficit Hyperactivity Disorder (ADHD). *Journal of Attention Disorders*, *28*(7), 1045–1062. https://doi.org/10.1177/10870547241226654

Hanscombe, K. B., Trzaskowski, M., Haworth, C. M. A., Davis, O. S. P., Dale, P. S., & Plomin, R. (2012). Socioeconomic Status (SES) and Children’s Intelligence (IQ): In a UK-Representative Sample SES Moderates the Environmental, Not Genetic, Effect on IQ. *PLOS ONE*, *7*(2), e30320. https://doi.org/10.1371/journal.pone.0030320

Haworth, C. M. A., Harlaar, N., Kovas, Y., Davis, O. S. P., Oliver, B. R., Hayiou-Thomas, M. E., Frances, J., Busfield, P., McMillan, A., Dale, P. S., & Plomin, R. (2007). Internet Cognitive Testing of Large Samples Needed in Genetic Research. *Twin Research and Human Genetics*, *10*(4), 554–563. https://doi.org/10.1375/twin.10.4.554

Lecrubier, Y., Sheehan, D., Weiller, E., Amorim, P., Bonora, I., Harnett Sheehan, K., Janavs, J., & Dunbar, G. (1997). The Mini International Neuropsychiatric Interview (MINI). A short diagnostic structured interview: Reliability and validity according to the CIDI. *European Psychiatry*, *12*(5), 224–231. https://doi.org/10.1016/S0924-9338(97)83296-8

Löwe, B., Decker, O., Müller, S., Brähler, E., Schellberg, D., Herzog, W., & Herzberg, P. Y. (2008). Validation and Standardization of the Generalized Anxiety Disorder Screener (GAD-7) in the General Population. *Medical Care*, *46*(3), 266. https://doi.org/10.1097/MLR.0b013e318160d093

Martin, A., Rief, W., Klaiberg, A., & Braehler, E. (2006). Validity of the Brief Patient Health Questionnaire Mood Scale (PHQ-9) in the general population. *General Hospital Psychiatry*, *28*(1), 71–77. https://doi.org/10.1016/j.genhosppsych.2005.07.003

Pettersson, R., Söderström, S., & Nilsson, K. W. (2018). Diagnosing ADHD in Adults: An Examination of the Discriminative Validity of Neuropsychological Tests and Diagnostic Assessment Instruments. *Journal of Attention Disorders*, *22*(11), 1019–1031. https://doi.org/10.1177/1087054715618788

Ramos-Quiroga, J. A., Nasillo, V., Richarte, V., Corrales, M., Palma, F., Ibáñez, P., Michelsen, M., Van de Glind, G., Casas, M., & Kooij, J. J. S. (2019). Criteria and Concurrent Validity of DIVA 2.0: A Semi-Structured Diagnostic Interview for Adult ADHD. *Journal of Attention Disorders*, *23*(10), 1126–1135. https://doi.org/10.1177/1087054716646451

Raven, J. C. (1960). *Guide to the Standard Progressive Matrices*. London: H. K. Lewis.

Raven, J. C. (1962). *Extended Guide to using the Mill Hill Vocabulary Scale with the Progressive Matrices Scales*. London: H. K. Lewis.

White, I. R., Royston, P., & Wood, A. M. (2011). Multiple imputation using chained equations: issues and guidance for practice. Statistics in medicine, 30(4), 377-399.

Saleh, A., Fuchs, C., Taylor, W. D., & Niarhos, F. (2018). Evaluating the consistency of scales used in adult attention deficit hyperactivity disorder assessment of college-aged adults. *Journal of American College Health*, *66*(2), 98–105. https://doi.org/10.1080/07448481.2017.1377206

Tye, C., Rijsdijk, F., Greven, C. U., Kuntsi, J., Asherson, P., & McLoughlin, G. (2012). Shared genetic influences on ADHD symptoms and very low-frequency EEG activity: A twin study. *Journal of Child Psychology and Psychiatry*, *53*(6), 706–715. https://doi.org/10.1111/j.1469-7610.2011.02501.x

von Stumm, S., & Plomin, R. (2015). Socioeconomic status and the growth of intelligence from infancy through adolescence. *Intelligence*, *48*, 30–36. https://doi.org/10.1016/j.intell.2014.10.002

World Health Organization. (1996). WHOQOL-BREF : introduction, administration, scoring and generic version of the assessment World Health Organization. In: World Health Organization.
